# Supplementary material for: Etiological, epidemiological, and clinical features of acute diarrhea in China
Source: Nat Commun. 2021 Apr 29;12:2464. doi: 10.1038/s41467-021-22551-z (PMC8085116; doi:10.1038/s41467-021-22551-z)
Supplement: Supplementary file 1 — Supplementary Information [file 41467_2021_22551_MOESM1_ESM.pdf]

Supplementary Information

Etiological, epidemiological, and clinical features of acute diarrhea in China

Supplementary Methods

The sentinel hospitals and reference laboratories

In 2009, an active surveillance for patients with diarrhea was performed in 31 provinces (autonomous regions or municipalities) of China. The guideline was adhered to when selecting sentinel hospitals: include children's, general, urban, and rural community health service centers[1]. A national surveillance protocol including guidelines for patient enrollment, specimen collection and laboratory testing, case reporting form and protocols for data transmission and related standard operating procedures (SOP) in Chinese were developed by Chinese Center for Disease Control and Prevention (China CDC, Beijing)[2]. All the sentinel hospitals had undergone training to be qualified for recruiting patients, sample collection and test following the SOP, before the surveillance was started. The reference laboratories were responsible for conducting comprehensive pathogenic tests on the specimens.

Definition of seven ecological regions

Based on previous criteria, seven ecological regions were defined to perform geographical related analysis: northeast China district, north China district, Inner Mongolia-Xinjiang district, Qinghai-Tibet district, southwest China district, central China district and south China district[3]. Different climatic types dominated, with most of the northeast and north China district having temperate monsoon climate, Inner Mongolia-Xinjiang district having temperate continental climate, Qinghai-Tibet district having plateau mountain climate, southwest and central China having subtropical monsoon climate, south China district having subtropical monsoon climate and tropical monsoon climate.

Data were delimited by latitude. Studies conducted above 40° having temperate continental climate, those between 30° and 40° having temperate monsoon climate and subtropical monsoon climate, and those below 30° having subtropical monsoon climate and tropical monsoon climate.

Statistical Analysis

Patients were classified into urban and rural, depending on the administrative classification of their residence address[4]. According to the average monthly temperature of the sentinel city, the top six were classified as "warm months", while the rest were "cold months". For all the analysis in the current study, frequencies and rates were estimated for categorical variables while medians and interquartile ranges (IQRs) were estimated for continuous variables with abnormal distribution. Pearson chi-square test or Fisher exact test was performed to compare the categorical variables. Parasites are not studied for their interaction with virus or bacteria due to only small number of samples were tested for parasites.

Joinpoint regression model was used to describe trends data and fits a series of joined linear models. The joinpoint regression model for the observations, (x_1, y_1), ..., (x_n, y_n), where $x_1 \leq ... \leq x_n$ without loss of generality, may be written as

$$E[y|x] = \beta_0+\beta_1 x+\delta_1(x-\tau_1)^{+}+\ldots+\delta_k(x-\tau_k)^{+}$$

where the τ_k 's are the unknown joinpoints and $\alpha^{+}=\alpha$ for $\alpha>0$ and 0 otherwise[5].

Linear trends were estimated by using annual percentage change (APC) which is calculated as $APC_i=[Exp(b_i)-1)]\times100$[6], where b_i represents the slope of the period segment. Average annual percent change (AAPC) is a summary measure of the trend over a prespecified fixed interval. It allows us to use a single number to describe the average APCs over a period of multiple years.

We infer signatures of virus-virus interactions from the nonrandom patterns of virus mixing

captured by coinfection information by assessing whether the propensity of a given virus X (explanatory) to coinfect with another virus Y (response) was higher, lower, or equal to the overall propensity of any (remaining) virus group to coinfect with virus Y. We adjusted for the effects of age (AGE), gender (SEX), and the time from onset to hospital admission (delay). To distinguish interactions between explanatory and response viruses from unrelated seasonal changes in infection risk, we also adjusted for the monthly background prevalence of response virus infections. To do so, we adjusted the total number of infections with the response virus (VCOUNT) and the total number tested (TCOUNT) within a 15-d window either side of each (earliest) sample collection date for each individual observation[7].

Each of the five virus models followed the following form where P_i^Y indicates the probability that individual i is coinfected with virus Y, and $X1 \ldots X4$ indicates the four explanatory viruses:

$$\log (P_i^Y / (1- P_i^Y)) = \beta 0 + \beta 1 AGE_i + \beta 2 SEX_i + \beta 3 delay_i + \beta 4 \log (V.COUNT_i + 1) + \beta 5 \log (T.COUNT_i + 1) + \beta 6 X1_i + \beta 7 X2_i + \beta 8 X3_i + \beta 9 X4_i.$$

Statistical analysis about the diagnostic tree to distinguish between bacterial and viral infections

In this paper, the binary eXtreme Gradient Boosting (XGBoost) model was taken to differentiate viral infections and bacterial infections. All the data (5,816 patients with viral single infections and 2,942 patients with bacterial single infections) were divided into training set and test set with 80% and 20% proportion in each one and the best fit of the model according to Bayesian Optimization. The K fold Cross-validation were used in the training data in order to choose the better parameters and avoid the over fitting to some extent. The relative contributions of variables to the virus infections were estimated. We also created a logistic regression model to distinguish the bacterial and viral infections based on the 11 variables regarding demographical and clinical symptoms/syndrome, and the odds ratio (OR) of them and 95% confidence interval (CI) were estimated to display the effects of them on viral infections.

Meteorological Variable obtaining and Usage

The association between daily pathogen positive rates and six meteorological parameters (including daily average temperature, daily average relative humidity, daily sunshine duration, daily precipitation, daily average wind speed and daily average air pressure) and four socioeconomic factors (GDP *per capita*, population density, proportion of children and proportion of the older people) were explored by using a negative binomial regression model at the sentinel city level. The analysis included delays of 0 to 10 days to determine the best fit of the model according to Akaike Information Criteria. For incidence rate ratio (IRR) and 95% CI estimation, the difference in average daily temperature is 5 °C, the difference in average daily relative humidity is 10%, the difference in duration of sunshine is 10 hours, the difference in daily precipitation is 10 mm, the difference in daily wind speed is 1m/s and the difference in daily pressure is 10 kPa. For socioeconomic factors, the per capita GDP difference is 10,000 yuan, the population density difference is 1,000 people/km^2, the proportion of children and proportion of the older people is 1%. Data of meteorological factors was extracted from the website of National Meteorological Information Center of China (data.cma.cn) and the data of socioeconomic factors was extracted from the website of National Bureau of Statistics of China (www.stats.gov.cn).

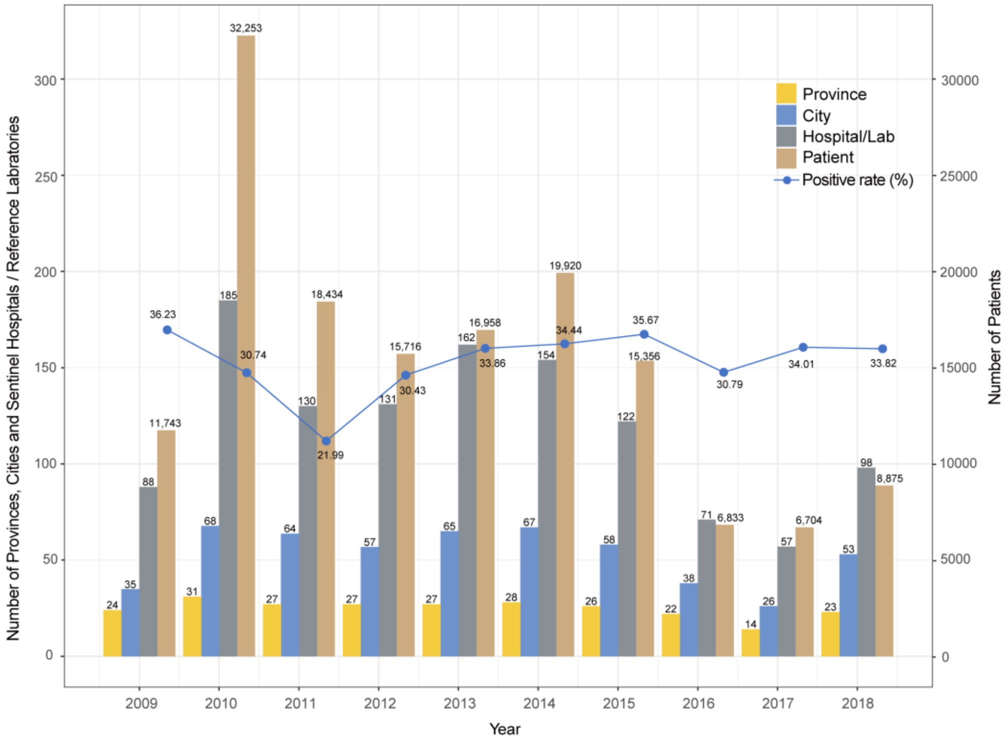

Supplementary Fig. 1: Annual number of participating provinces, sentinel cities, sentinel hospitals/reference laboratories, patients with acute diarrhea and positive rate included in this study, 2009–2018.

The positive rate was calculated by dividing the number of at least one positive pathogen by the at least one number tested for pathogen. Source data are provided as a Source Data file.

Supplementary Table 1: Positive rate and the time from onset to hospital admission stratified by age in patients with diarrhea.

	All cases	Age group					p
		<5 year	5–17 year	18–45 year	46–59 year	≥60 year	
All enteropathogen tested	N=3,330	N=1,059	N=222	N=996	N=396	N=657	-
Positive rate*	868 (26.07)	636 (60.06)	59 (26.58)	78 (7.83)	36 (9.09)	59 (8.98)	<0.0001
Delay♯ (IQR), day	1 (1–2)	3 (1–3)	1 (1–1)	1 (1–1)	1 (1–2)	1 (1–1)	<0.0001

Data are presented as the N, the detected case number, (% as positive rate) unless otherwise indicated. The positive rate was calculated by dividing the number of at least one positive pathogen by the number of all enteropathogens tested. *Chi square test for comparisons among patients positive for different groups. ♯Wilcoxon test for comparisons among patients positive for different groups. All statistical tests were two-sided and $p<0.05$ was statistically significant. Delay means the time from onset to hospital admission. IQR=interquartile range.

Supplementary Table 2: Demographic characteristics compared between patients with acute diarrhea with enteropathogen positive or enteropathogen negative detection.

Characteristics	Enteropathogen positive	Enteropathogen negative	*p*
Sex[*]			0.12
Male	533 (61.41)	1,437 (58.37)	
Female	335 (38.59)	1,025 (41.63)	
Age[#] (IQR), year	1 (0.83–5)	35 (19–59)	<0.0001
Age group[*]			<0.0001
<5 year	636 (73.27)	423 (17.18)	
5–17 year	59 (6.80)	163 (6.62)	
18–45 year	78 (8.99)	918 (37.29)	
46–59 year	36 (4.15)	360 (14.62)	
≥60 year	59 (6.80)	598 (24.29)	
Delay[#] (IQR), day	3 (1–3)	1 (1–2)	<0.0001
Ecological Regions[*]			<0.0001
Northeast China	-	-	
North China	12 (1.38)	218 (8.85)	
Inner Mongolia-Xinjiang	-	-	
Qinghai-Tibet	-	-	
Southwest China	80 (9.22)	1,114 (45.25)	
Central China	770 (88.71)	1,112 (45.17)	
South China	6 (0.69)	18 (0.73)	
Residence[*]			<0.0001
Urban	705 (81.22)	1,538 (62.47)	
Rural	127 (14.63)	722 (29.33)	

Data are n (%) unless otherwise indicated. All enteropathogens tested cases were used to compare enteropathogen positive and enteropathogen negative cases. Enteropathogen positive cases were defined as any enteropathogen positive detection. Enteropathogen negative cases were defined as all enteropathogens negative detection. [*]Chi square test for comparisons when enteropathogen positive *vs* enteropathogen negative cases. [#]Wilcoxon test for comparisons when enteropathogen positive *vs* enteropathogen negative cases. All statistical tests were two-sided and $p<0.05$ was statistically significant. Delay means the time from onset to hospital admission. IQR=interquartile range.

Supplementary Table 3: The pathogen spectrum of viruses, bacteria, and parasites for patients with diarrhea, in the mainland of China, 2009–2018.

	All cases		<6 month		6–11 month		1–4 year		5–17 year		18–45 year		46–59 year		≥60 year	
All viruses tested	N=58,620		N=6,779		N=9,632		N=13,982		N=3,169		N=12,997		N=5,621		N=6,440	
Positive rate[†]	18,676 (31.86)		2,180 (32.16)		4,560 (47.34)		6,410 (45.84)		820 (25.88)		2,415 (18.58)		1,121 (19.94)		1,170 (18.17)	
Viruses	Rank	Proportion	Rank	Proportion	Rank	Proportion	Rank	Proportion	Rank	Proportion	Rank	Proportion	Rank	Proportion	Rank	Proportion
Rotavirus A	1	9,312 (45.05)	1	1,257 (50.60)	1	2,847 (55.92)	1	3,822 (52.99)	2	306 (34.65)	2	502 (19.58)	2	286 (24.14)	2	292 (23.32)
Norovirus	2	6,658 (32.21)	2	583 (23.47)	2	1,237 (24.30)	2	1,738 (24.10)	1	311 (35.22)	1	1,451 (56.59)	1	660 (55.70)	1	678 (54.15)
Adenovirus	3	1,916 (9.27)	3	299 (12.04)	3	483 (9.49)	3	786 (10.90)	3	106 (12.00)	5	120 (4.68)	5	53 (4.47)	4	69 (5.51)
Astrovirus	4	1,485 (7.18)	4	231 (9.30)	4	285 (5.60)	4	420 (5.82)	4	99 (11.21)	3	255 (9.95)	3	85 (7.17)	3	110 (8.79)
Sapovirus	5	940 (4.55)	5	64 (2.58)	5	171 (3.36)	5	327 (4.53)	5	44 (4.98)	4	187 (7.29)	4	80 (6.75)	5	67 (5.35)
Rotavirus B	7	179 (0.87)	7	24 (0.97)	6	35 (0.69)	6	72 (1.00)	6	11 (1.25)	7	14 (0.55)	6	11 (0.93)	7	12 (0.96)
Rotavirus C	6	182 (0.88)	6	26 (1.05)	7	33 (0.65)	7	48 (0.67)	7	6 (0.68)	6	35 (1.37)	7	10 (0.84)	6	24 (1.92)
All Bacteria tested	N=59,384		N=5,711		N=7,149		N=11,201		N=3,570		N=16,135		N=7,203		N=8,415	
Positive rate[&]	10,825 (18.23)		521 (9.12)		1,060 (14.83)		2,217 (19.79)		709 (19.86)		3,547 (21.98)		1,367 (18.98)		1,404 (16.68)	
Bacteria	Rank	Proportion	Rank	Proportion	Rank	Proportion	Rank	Proportion	Rank	Proportion	Rank	Proportion	Rank	Proportion	Rank	Proportion
DEC	1	4,932 (43.31)	1	249 (46.89)	2	473 (42.35)	1	913 (38.97)	1	301 (41.06)	1	1,693 (44.93)	1	645 (45.01)	1	658 (44.98)
NTS	2	2,859 (25.11)	2	173 (32.58)	1	490 (43.87)	2	864 (36.88)	2	158 (21.56)	3	587 (15.58)	2	284 (19.82)	2	303 (20.71)
Shigella	4	783 (6.88)	4	32 (6.03)	4	45 (4.03)	3	315 (13.44)	3	115 (15.69)	5.5	137 (3.64)	6	64 (4.47)	6	75 (5.13)
V. parahaemolyticus	3	1,233 (10.83)	5	13 (2.45)	8.5	5 (0.45)	7	18 (0.77)	5	36 (4.91)	2	778 (20.65)	3	225 (15.70)	3	158 (10.80)
A. hydrophila	6	453 (3.98)	3	37 (6.97)	3	47 (4.21)	5	61 (2.60)	6	22 (3.00)	5.5	137 (3.64)	4	68 (4.75)	4	81 (5.54)
C. jejuni	5	516 (4.53)	6	12 (2.26)	5	27 (2.42)	4	98 (4.18)	4	54 (7.37)	4	180 (4.78)	5	65 (4.54)	5	80 (5.47)
P. shigelloides	7	255 (2.24)	-	0 (0.00)	7	7 (0.63)	9	7 (0.30)	9	10 (1.36)	7	130 (3.45)	7	48 (3.35)	7	53 (3.62)
V. cholerae	10	70 (0.61)	-	0 (0.00)	-	0 (0.00)	10	5 (0.21)	10.5	3 (0.41)	8	46 (1.22)	10	6 (0.42)	10	10 (0.68)
Y. enterocolitica	8	146 (1.28)	7	11 (2.07)	6	14 (1.25)	6	44 (1.88)	7	16 (2.18)	10	29 (0.77)	9	11 (0.77)	8	21 (1.44)
C. coli	9	97 (0.85)	8	3 (0.56)	8.5	5 (0.45)	8	14 (0.60)	8	15 (2.05)	9	31 (0.82)	8	12 (0.84)	9	17 (1.16)
V. fluvialis	11	34 (0.30)	-	0 (0.00)	10	3 (0.27)	12.5	1 (0.04)	10.5	3 (0.41)	11	18 (0.48)	11	3 (0.21)	11	6 (0.41)
V. mimicus	12	7 (0.06)	9	1 (0.19)	11	1 (0.09)	12.5	1 (0.04)	-	0 (0.00)	12	2 (0.05)	12.5	1 (0.07)	12	1 (0.07)
Y. pseudotuberculosis	13	3 (0.03)	-	0 (0.00)	-	0 (0.00)	11	2 (0.09)	-	0 (0.00)	-	0 (0.00)	12.5	1 (0.07)	-	0 (0.00)
All parasites tested	N=11,167		N=828		N=1,122		N=2,077		N=690		N=3,130		N=1,444		N=1,876	
Positive rate[*]	192 (1.72)		5 (0.60)		6 (0.53)		16 (0.77)		16 (2.32)		73 (2.33)		45 (3.12)		31 (1.65)	
Parasites	Rank	Proportion	Rank	Proportion	Rank	Proportion	Rank	Proportion	Rank	Proportion	Rank	Proportion	Rank	Proportion	Rank	Proportion
E. histolytica	1	108 (55.38)	1.5	2 (40.00)	1	3 (50.00)	1	8 (47.06)	1	10 (62.50)	1	42 (57.53)	1	27 (58.70)	1	16 (50.00)
G. lamblia	2	64 (32.82)	3	1 (20.00)	3	1 (16.67)	2	7 (41.18)	2	4 (25.00)	2	24 (32.88)	2	14 (30.43)	2	13 (40.63)
Cryptosporidium	3	23 (11.79)	1.5	2 (40.00)	2	2 (33.33)	3	2 (11.76)	3	2 (12.50)	3	7 (9.59)	3	5 (10.87)	3	3 (9.38)

Overall positive rate	868 (26.07)	87 (46.77)	187 (60.13)	362 (64.41)	59 (26.58)	78 (7.83)	36 (9.09)	59 (8.98)

The viral spectrum and its subgroups are shown based on 58,620 cases with all seven viruses tested. The bacterial spectrum and its subgroups are displayed based on 59,384 cases with all 13 bacteria tested. The parasitical spectrum and its subgroups are displayed based on 11,167 cases with all three parasites tested.

Data are presented as the N, the case number, (% as proportion) unless otherwise indicated. The ranking was ordered based on the proportion.

†Viral positive rate denotes the rate of any virus positive cases in all viruses tested cases.

&Bacterial positive rate denotes the rate of any bacterium positive cases in all bacteria tested cases.

*Parasitical positive rate denotes the rate of any parasite positive cases in all parasites tested cases.

DEC=diarrheagenic *Escherichia coli*; NTS=nontyphoidal *Salmonella*.

Supplementary Table 4: Positive rate of viral, bacterial, and parasitical pathogens in patients with diarrhea in the mainland of China, 2009–2018.

	All cases (N, %)	Sex (N, %)		*p*	Age (N, %)							*p*
		Male	Female		<6 month	6–11 month	1–4 year	5–17 year	18–45 year	46–59 year	≥60 year	
Virus	28,811 (33.61)	17,804 (35.12)	11,007 (31.42)	-	3,634 (33.59)	6,874 (46.73)	9,890 (44.94)	1,361 (27.85)	3,734 (21.55)	1,607 (21.86)	1,711 (19.82)	-
Rotavirus A (N=74,307)	15,155 (20.40)	9,739 (21.94)	5,416 (18.11)	<0.0001	2,356 (23.54)	4,653 (33.72)	6,297 (31.53)	616 (14.91)	591 (4.30)	312 (5.29)	330 (4.90)	<0.0001
Norovirus (N=77,855)	9,707 (12.47)	5,744 (12.63)	3,963 (12.24)	0.11	821 (8.98)	1,554 (12.41)	2,362 (12.40)	444 (10.48)	2,406 (14.07)	1,021 (14.06)	1,099 (12.87)	<0.0001
GI[#]	849 (9.15)	482 (8.79)	367 (9.68)	0.14	56 (7.21)	114 (7.76)	169 (7.58)	48 (11.51)	276 (11.95)	100 (10.02)	86 (8.00)	<0.0001
GII[#]	8,426 (90.85)	5,002 (91.21)	3,424 (90.32)	-	721 (92.79)	1,356 (92.24)	2,060 (92.42)	369 (88.49)	2,033 (88.05)	898 (89.98)	989 (92.00)	-
Adenovirus (N=80,054)	2,669 (3.33)	1,642 (3.50)	1,027 (3.09)	0.0015	390 (4.09)	655 (4.97)	1,069 (5.36)	170 (3.90)	204 (1.19)	86 (1.18)	95 (1.10)	<0.0001
Astrovirus (N=79,529)	2,201 (2.77)	1,307 (2.81)	894 (2.71)	0.40	349 (3.69)	417 (3.20)	593 (3.00)	140 (3.24)	400 (2.34)	140 (1.92)	162 (1.89)	<0.0001
Sapovirus (N=77,436)	1,372 (1.77)	837 (1.85)	535 (1.66)	0.048	89 (0.98)	225 (1.81)	453 (2.39)	74 (1.76)	307 (1.81)	123 (1.70)	101 (1.19)	<0.0001
Rotavirus B (N=71,917)	311 (0.43)	201 (0.48)	110 (0.36)	0.017	43 (0.54)	63 (0.57)	147 (0.87)	12 (0.30)	18 (0.11)	13 (0.18)	15 (0.18)	<0.0001
Rotavirus C (N=71,567)	258 (0.36)	180 (0.43)	78 (0.26)	0.0001	33 (0.42)	48 (0.44)	99 (0.59)	7 (0.17)	35 (0.21)	12 (0.17)	24 (0.29)	<0.0001
Bacterium	16,841 (16.82)	9,432 (16.43)	7,409 (17.34)	-	756 (7.92)	1,515 (12.97)	3,311 (17.93)	1,190 (19.77)	5,802 (20.27)	2,176 (17.63)	2,091 (15.55)	-
DEC (N=92,238)	6,191 (6.71)	3,427 (6.50)	2,764 (6.99)	0.0032	323 (3.67)	590 (5.52)	1,113 (6.55)	375 (6.85)	2,191 (8.33)	790 (6.90)	809 (6.46)	<0.0001
EAEC[#]	1,764 (28.51)	969 (28.28)	795 (28.80)	0.0015	122 (37.89)	228 (38.64)	403 (36.24)	94 (25.13)	509 (23.23)	184 (23.32)	224 (27.69)	<0.0001
EPEC[#]	1,569 (25.36)	893 (26.06)	676 (24.49)	-	83 (25.78)	182 (30.85)	395 (35.52)	97 (25.94)	482 (22.00)	168 (21.29)	162 (20.02)	-
ETEC[#]	1,489 (24.07)	773 (22.56)	716 (25.94)	-	48 (14.91)	84 (14.24)	149 (13.40)	80 (21.39)	633 (28.89)	255 (32.32)	240 (29.67)	-
EIEC[#]	408 (6.59)	252 (7.35)	156 (5.65)	-	31 (9.63)	36 (6.10)	75 (6.74)	36 (9.63)	141 (6.44)	40 (5.07)	49 (6.06)	-
EHEC[#]	187 (3.02)	117 (3.41)	70 (2.54)	-	29 (9.01)	42 (7.12)	40 (3.60)	7 (1.87)	37 (1.69)	14 (1.77)	18 (2.22)	-
DEC-untyped	770 (12.45)	423 (12.34)	347 (12.57)	-	9 (2.80)	18 (3.05)	50 (4.50)	60 (16.04)	389 (17.75)	128 (16.22)	116 (14.34)	-
NTS (N=99,114)	4,366 (4.41)	2,481 (4.37)	1,885 (4.46)	0.50	253 (2.67)	681 (5.87)	1,214 (6.67)	261 (4.44)	1,033 (3.64)	474 (3.87)	450 (3.37)	<0.0001
Shigella (N=95,593)	2,331 (2.44)	1,366 (2.50)	965 (2.35)	0.14	67 (0.74)	123 (1.11)	717 (4.03)	333 (5.80)	616 (2.26)	228 (1.93)	247 (1.91)	<0.0001
V. parahaemolyticus (N=95,155)	1,981 (2.08)	1,031 (1.90)	950 (2.33)	<0.0001	23 (0.25)	14 (0.13)	33 (0.19)	60 (1.06)	1,238 (4.52)	372 (3.14)	241 (1.86)	<0.0001
A. hydrophila (N=91,718)	908 (0.99)	497 (0.95)	411 (1.04)	0.16	57 (0.66)	72 (0.70)	126 (0.76)	40 (0.74)	311 (1.17)	151 (1.31)	151 (1.19)	<0.0001
C. jejuni (N=90,391)	788 (0.87)	463 (0.90)	325 (0.84)	0.33	25 (0.29)	57 (0.55)	146 (0.88)	76 (1.41)	276 (1.07)	92 (0.81)	116 (0.95)	<0.0001
P. shigelloides (N=90,874)	453 (0.50)	238 (0.46)	215 (0.55)	0.057	6 (0.07)	12 (0.12)	16 (0.10)	20 (0.37)	237 (0.90)	84 (0.74)	78 (0.63)	<0.0001
V. cholerae (N=96,949)	301 (0.31)	157 (0.28)	144 (0.35)	0.074	3 (0.03)	8 (0.07)	24 (0.14)	21 (0.36)	161 (0.58)	45 (0.37)	39 (0.30)	<0.0001
Y. enterocolitica (N=93,813)	282 (0.30)	167 (0.31)	115 (0.29)	0.50	17 (0.19)	24 (0.22)	76 (0.44)	26 (0.46)	75 (0.28)	26 (0.22)	38 (0.30)	0.0004
C. coli (N=89,049)	131 (0.15)	77 (0.15)	54 (0.14)	0.67	3 (0.03)	11 (0.11)	24 (0.15)	18 (0.34)	38 (0.15)	17 (0.15)	20 (0.17)	0.0011
V. fluvialis (N=61,673)	44 (0.07)	26 (0.08)	18 (0.07)	0.67	0 (0.00)	3 (0.04)	1 (0.01)	5 (0.14)	23 (0.14)	6 (0.08)	6 (0.07)	0.0007
V. mimicus (N=61,676)	8 (0.01)	5 (0.01)	3 (0.01)	1.0	1 (0.02)	1 (0.01)	1 (0.01)	0 (0.00)	3 (0.02)	1 (0.01)	1 (0.01)	0.96

Y. pseudotuberculosis (N=60,951)	3 (0.005)	2 (0.01)	1 (0.004)	1.0	0 (0.00)	0 (0.00)	2 (0.02)	0 (0.00)	0 (0.00)	1 (0.01)	0 (0.00)	0.31
Parasite	253 (1.95)	155 (2.06)	98 (1.79)	-	5 (0.57)	9 (0.72)	19 (0.83)	17 (2.20)	112 (2.99)	55 (3.09)	36 (1.59)	-
E. histolytica (N=11,861)	125 (1.05)	76 (1.09)	49 (1.00)	0.61	2 (0.23)	5 (0.41)	10 (0.45)	10 (1.33)	52 (1.59)	30 (1.97)	16 (0.80)	<0.0001
G. lamblia (N=12,433)	97 (0.78)	56 (0.78)	41 (0.78)	0.98	1 (0.12)	1 (0.09)	7 (0.32)	5 (0.68)	45 (1.23)	20 (1.16)	18 (0.83)	<0.0001
Cryptosporidium (N=12,625)	34 (0.27)	26 (0.36)	8 (0.15)	0.028	2 (0.23)	3 (0.24)	3 (0.13)	2 (0.27)	15 (0.41)	6 (0.35)	3 (0.14)	0.41

Data are presented as the N, the detected case number, (% as positive rate) unless otherwise indicated. Chi square test or Fisher's exact test for comparisons among patients positive for different groups. All statistical tests were two-sided and $p<0.05$ was statistically significant. [#]indicated the proportion of each type within the positive detection. For subtypes of Norovirus and DEC, denominator is the number of positive-detection pathogens used for further isolation and the numerator is the number of positive detection of subtypes.

DEC=diarrheagenic *Escherichia coli*; NTS=nontyphoidal *Salmonella*; EAEC=enteroaggregative *E. coli*; EPEC=enteropathogenic *E. coli*; ETEC=enterotoxigenic *E. coli*; EIEC=enteroinvasive *E. coli*; EHEC=enterohaemorrhagic *E. coli*.

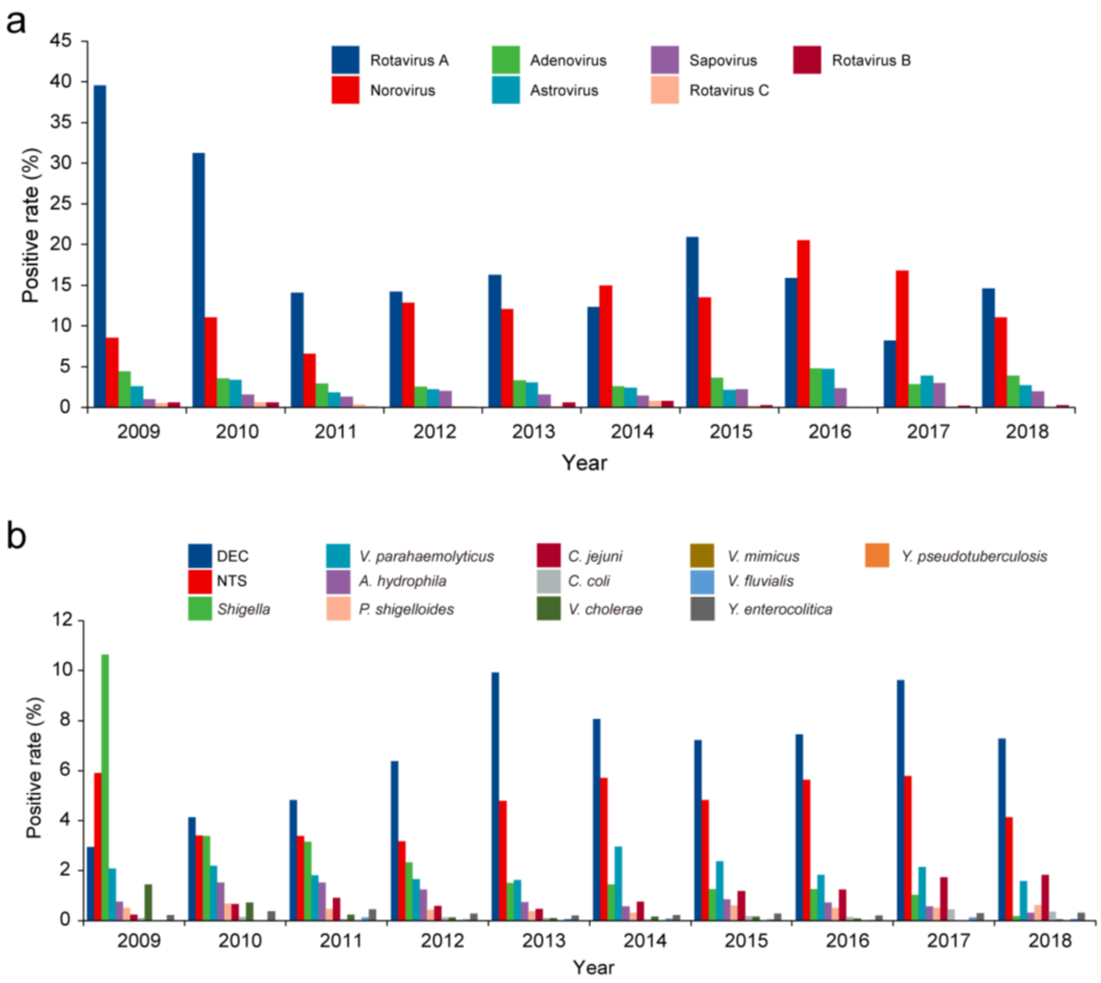

Supplementary Fig. 2: Annual positive rate of diarrheal pathogens for seven viruses and thirteen bacteria among patients with diarrhea in the mainland of China, 2009–2018.
(a) Positive rate of viruses. (b) Positive rate of bacteria. DEC=diarrheagenic *Escherichia coli*; NTS=nontyphoidal *Salmonella*. Source data are provided as a Source Data file.

Supplementary Table 5: Average annual percentage change in positive rate of enteropathogens, in the mainland of China, 2009–2018.

	Trend of positive rate	Average annual percentage change (95% CI)	*p*
Rotavirus A	Decrease	-12.4% (-20.3 to -3.7)	0.0014
Norovirus	Stable	6.0% (-0.5 to 12.9)	0.1
Adenovirus	Stable	0.1% (-5.4 to 5.9)	1.0
Astrovirus	Stable	1.2% (-6.0 to 8.9)	0.69
Sapovirus	Increase	6.6% (0.9 to 12.6)	0.0069
Rotavirus B	Stable	-4.1% (-21.1 to 16.5)	0.62
Rotavirus C[†]	Stable	-2.5% (-23.4 to 24.1)	0.84
DEC	Increase	10.7% (4.3 to 17.6)	0.00097
NTS	Stable	4.5% (-2.0 to 11.5)	0.11
Shigella	Decrease	-26.5% (-35.0 to -16.8)	<0.0001
V. parahaemolyticus	Stable	0.7% (-6.1 to 8.0)	0.84
A. hydrophila	Decrease	-14.2% (-21.1 to -6.8)	<0.0001
C. jejuni	Increase	14.5% (6.1 to 23.6)	<0.0001
P. shigelloides	Stable	-1.3% (-8.5 to 6.5)	0.69
V. cholerae	Decrease	-26.0% (-38.9 to -10.5)	0.0019
Y. enterocolitica	Stable	-4.8% (-11.8 to 2.7)	0.13
C. coli	Increase	17.5% (4.4 to 32.4)	0.0019
G. lamblia[†]	Decrease	-30.5% (-40.7 to -18.5)	<0.0001
E. histolytica[†]	Decrease	-37.4% (-49.5 to -22.5)	<0.0001
Cryptosporidium[†]	Stable	-27.2% (-76.9 to 128.9)	0.62

A normal (Z) distribution was used to assess significance of the average annual percentage change, and the parametric method was used to calculate 95% CI. All statistical tests were two-sided and $p<0.05$ was statistically significant. [†]When positive rate data contained zero, we substitute the zero with 1% of the smallest positive rate[8]. DEC=diarrheagenic *Escherichia coli*; NTS=nontyphoidal *Salmonella*; 95% CI=95% confidence interval.

Supplementary Table 6: Frequency of Rotavirus A genotypes identified in patients with diarrhea in the mainland of China, 2009–2018.

Genotypes	P[4]	P[6]	P[8]	P[9]	P[10]	P[11]	P Mix	P non-typable	Others	Total
G1	105	2	519	5	14	28	25	13	9	720
G2	183	1	42	1	15	6	12	11	8	279
G3	71	2	651	22	31	17	42	23	2	861
G4	25	1	8	3	6	1	14	3	2	63
G8	5	3	31	9	1	0	1	4	0	54
G9	36	12	1,117	6	14	44	12	71	31	1,343
G Mix	67	4	84	2	0	2	43	5	5	212
G non-typable	13	10	30	3	4	5	1	3,785	0	3,851
Others	3	0	30	0	2	115	1	1	69	221
Total	508	35	2,512	51	87	218	151	3,916	126	7,604

Data are presented as the case number in each genotype.

Supplementary Table 7: Positive rate of specific pathogen between male and female stratified by age in patients with diarrhea, 2009–2018.

	<5 year (N=43,778)		*p*	5–17 year (N=4,132)		*p*	18–45 year (N=13,760)		*p*	46–59 year (N=5,902)		*p*	≥60 year (N=6,735)		*p*
	Male	Female		Male	Female		Male	Female		Male	Female		Male	Female	
Viral positive rate															
Rotavirus A	8,744 (30.96)	4,562 (29.36)	0.0005	403 (15.35)	213 (14.13)	0.29	293 (3.99)	298 (4.65)	0.056	135 (4.73)	177 (5.81)	0.066	164 (4.93)	166 (4.87)	0.91
Norovirus	3,057 (11.77)	1,680 (11.40)	0.27	283 (10.60)	161 (10.29)	0.76	1,348 (14.63)	1,058 (13.41)	0.021	501 (14.50)	520 (13.66)	0.30	555 (13.34)	544 (12.43)	0.21
Adenovirus	1,334 (4.89)	780 (5.07)	0.43	104 (3.79)	66 (4.07)	0.65	106 (1.15)	98 (1.24)	0.61	50 (1.44)	36 (0.94)	0.046	48 (1.14)	47 (1.07)	0.73
Astrovirus	884 (3.27)	475 (3.11)	0.36	95 (3.50)	45 (2.79)	0.21	185 (2.02)	215 (2.72)	0.0023	69 (1.99)	71 (1.86)	0.67	74 (1.77)	88 (2.00)	0.43
Sapovirus	511 (1.98)	256 (1.75)	0.10	48 (1.81)	26 (1.67)	0.74	166 (1.82)	141 (1.79)	0.91	61 (1.77)	62 (1.64)	0.66	51 (1.23)	50 (1.15)	0.73
Rotavirus B	168 (0.74)	85 (0.65)	0.34	5 (0.20)	7 (0.46)	0.14	13 (0.15)	5 (0.07)	0.12	7 (0.21)	6 (0.16)	0.66	8 (0.20)	7 (0.16)	0.72
Rotavirus C	129 (0.57)	51 (0.39)	0.020	7 (0.28)	0 (0.00)	0.051	24 (0.27)	11 (0.14)	0.081	5 (0.15)	7 (0.19)	0.68	15 (0.37)	9 (0.21)	0.18
Bacterial positive rate															
DEC	1,285 (5.61)	741 (5.45)	0.52	227 (6.59)	148 (7.30)	0.31	1,148 (7.96)	1,043 (8.78)	0.017	387 (6.83)	403 (6.96)	0.77	380 (6.06)	429 (6.87)	0.063
NTS	1,306 (5.27)	842 (5.80)	0.026	153 (4.15)	108 (4.91)	0.17	536 (3.45)	497 (3.87)	0.064	256 (4.19)	218 (3.56)	0.069	230 (3.41)	220 (3.32)	0.77
Shigella	560 (2.35)	347 (2.47)	0.45	199 (5.53)	134 (6.25)	0.25	346 (2.33)	270 (2.18)	0.39	126 (2.16)	102 (1.71)	0.077	135 (2.09)	112 (1.73)	0.14
V. parahaemolyticus	47 (0.20)	23 (0.17)	0.47	43 (1.22)	17 (0.80)	0.14	685 (4.59)	553 (4.44)	0.56	158 (2.69)	214 (3.59)	0.0054	98 (1.51)	143 (2.22)	0.0030
A. hydrophila	161 (0.72)	94 (0.72)	0.97	22 (0.65)	18 (0.88)	0.33	178 (1.23)	133 (1.10)	0.34	66 (1.16)	85 (1.46)	0.16	70 (1.10)	81 (1.28)	0.36
C. jejuni	139 (0.62)	89 (0.68)	0.49	50 (1.48)	26 (1.28)	0.55	171 (1.22)	105 (0.89)	0.011	46 (0.83)	46 (0.80)	0.89	57 (0.94)	59 (0.96)	0.94
P. shigelloides	14 (0.06)	20 (0.15)	0.0086	19 (0.56)	1 (0.05)	0.0026	130 (0.91)	107 (0.90)	0.90	34 (0.60)	50 (0.86)	0.10	41 (0.66)	37 (0.59)	0.62
V. cholerae	15 (0.06)	20 (0.14)	0.013	11 (0.30)	10 (0.46)	0.33	90 (0.59)	71 (0.56)	0.73	20 (0.34)	25 (0.41)	0.48	21 (0.32)	18 (0.28)	0.66
Y. enterocolitica	74 (0.32)	43 (0.31)	0.99	15 (0.43)	11 (0.53)	0.59	45 (0.31)	30 (0.25)	0.35	11 (0.19)	15 (0.25)	0.46	22 (0.35)	16 (0.25)	0.33
C. coli	21 (0.09)	17 (0.13)	0.32	11 (0.33)	7 (0.35)	0.91	25 (0.18)	13 (0.11)	0.16	13 (0.24)	4 (0.07)	0.024	7 (0.12)	13 (0.21)	0.20
V. fluvialis	4 (0.03)	0 (0.00)	0.31	4 (0.17)	1 (0.07)	0.66	11 (0.12)	12 (0.15)	0.59	4 (0.11)	2 (0.05)	0.44	3 (0.07)	3 (0.07)	1.0
V. mimicus	2 (0.01)	1 (0.01)	1.0	0 (0.00)	0 (0.00)	-	2 (0.02)	1 (0.01)	1.0	0 (0.00)	1 (0.03)	1.0	1 (0.02)	0 (0.00)	0.48
Y. pseudotuberculosis	1 (0.01)	1 (0.01)	1.0	0 (0.00)	0 (0.00)	-	0 (0.00)	0 (0.00)	-	1 (0.03)	0 (0.00)	0.48	0 (0.00)	0 (0.00)	-
Parasitical positive rate															
E. histolytica	13 (0.47)	4 (0.25)	0.26	9 (1.91)	1 (0.35)	0.10	32 (1.75)	20 (1.39)	0.41	11 (1.42)	19 (2.53)	0.12	11 (0.96)	5 (0.58)	0.34
G. lamblia	9 (0.34)	0 (0.00)	0.030	3 (0.66)	2 (0.73)	1.0	28 (1.38)	17 (1.04)	0.36	9 (1.03)	11 (1.29)	0.61	7 (0.59)	11 (1.13)	0.17
Cryptosporidium	8 (0.29)	0 (0.00)	0.030	2 (0.43)	0 (0.00)	0.53	10 (0.50)	5 (0.31)	0.38	4 (0.46)	2 (0.24)	0.69	2 (0.17)	1 (0.10)	1.0

Data are n (%) unless otherwise indicated. Chi square test or Fisher's exact test for comparisons between male and female in different age groups. All statistical tests were two-sided and $p<0.05$ was statistically significant.
DEC=diarrheagenic *Escherichia coli*; NTS=nontyphoidal *Salmonella*.

Supplementary Table 8: Positive rate of specific pathogen compared between urban and rural patients with diarrhea, 2009–2018.

	Urban*	Rural*	*p*
Rotavirus A	11,477 (19.90)	3,185 (24.06)	<0.0001
Norovirus	7,935 (12.83)	1,439 (11.26)	<0.0001
Adenovirus	1,955 (3.07)	507 (3.87)	<0.0001
Astrovirus	1,625 (2.57)	468 (3.59)	<0.0001
Sapovirus	1,048 (1.71)	232 (1.82)	0.38
Rotavirus B	252 (0.43)	43 (0.42)	0.85
Rotavirus C	185 (0.32)	67 (0.65)	<0.0001
DEC	4,698 (6.76)	1,217 (6.57)	0.34
NTS	3,157 (4.24)	996 (4.94)	<0.0001
Shigella	1,101 (1.54)	1,136 (5.76)	<0.0001
V. parahaemolyticus	1,725 (2.4)	227 (1.2)	<0.0001
A. hydrophila	682 (0.98)	208 (1.15)	0.041
C. jejuni	733 (1.08)	45 (0.24)	<0.0001
P. shigelloides	404 (0.59)	41 (0.23)	<0.0001
V. cholerae	170 (0.23)	101 (0.51)	<0.0001
Y. enterocolitica	185 (0.26)	46 (0.24)	0.60
C. coli	124 (0.18)	6 (0.03)	<0.0001
V. fluvialis	37 (0.08)	7 (0.06)	0.51
V. mimicus	7 (0.02)	1 (0.01)	0.60
Y. pseudotuberculosis	3 (0.01)	0 (0.00)	0.39
E. histolytica	123 (1.23)	2 (0.13)	0.0001
G. lamblia	95 (0.9)	2 (0.13)	0.0013
Cryptosporidium	31 (0.29)	3 (0.19)	0.61

Data are n (%) unless otherwise indicated. *The number of patients in rural and urban areas did not equal the total number of patients due to missing data. Chi square test or Fisher's exact test for comparisons among patients positive for different groups. All statistical tests were two-sided and $p<0.05$ was statistically significant. DEC=diarrheagenic *Escherichia coli*; NTS=nontyphoidal *Salmonella*.

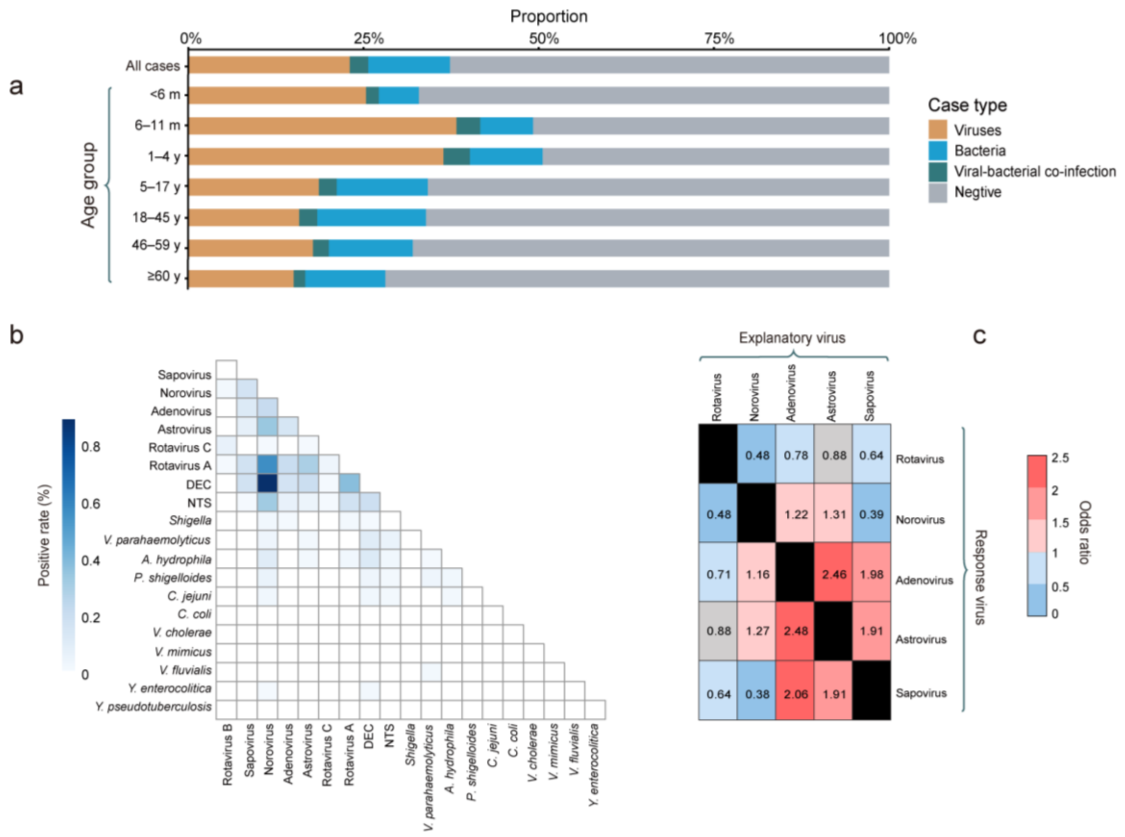

Supplementary Fig. 3: Viral-Bacterial co-infection and interaction of pathogens in patients with diarrhea, in the mainland of China, 2009–2018.
(a) Positive proportion of viruses, bacteria, and co-infections of viruses, bacteria from 25,239 patients with diarrhea who had all seven viruses and 13 bacteria tested. Co-infection indicates viral-bacterial co-infection. The length of colored bar indicates the positive rate. Orange bar means viral positive rate (viral mono-infection or viral-viral co-infection). Blue bar means bacterial positive rate (bacterial mono-infection or bacterial-bacterial co-infection). Green bar means viral-bacterial co-infection. Grey bar means none of the pathogens were positive. (b) Heatmap of the co-infection rate. (c) Odds ratio estimates from tests of 20 pairwise interactions generated from five viruses logistic regression models. We adjusted for the effects of age, gender, the time from onset to hospital admission and the monthly background prevalence of response virus infections. *P*-values corrected using Holm's method to account for multiple hypothesis testing. All statistical tests were two-sided and $p<0.05$ was statistically significant. The odds ratio, ranging from negative influence (blue) to positive (red). Gray indicates odds ratios without statistical significance. Rotavirus include rotavirus A, B and C. DEC=diarrheagenic *Escherichia coli*; NTS=nontyphoidal *Salmonella*. Source data are provided as a Source Data file.

Supplementary Table 9: Demographic and clinical features compared between patients with acute diarrhea with virus positive and bacterium positive detection.

	Overall Cases (N=8,758)			Children (<18 year) (N=4,458)			Adults (≥18 year) (N=4,300)		
	Virus positive (N=5,816)	Bacterium positive (N=2,942)	*p*	Virus positive (N=3,486)	Bacterium positive (N=972)	*p*	Virus positive (N=2,330)	Bacterium positive (N=1,970)	*p*
Baseline									
Age[♯] (IQR), year	2 (0.92–36)	29 (3–50)	<0.0001	1 (0.58–1)	1 (0.75–3)	<0.0001	47 (31–60)	40 (29–59)	<0.0001
Sex[*] (Male/Female)	3,369/2,447	1,545/1,397	<0.0001	2,173/1,313	592/380	0.42	1,196/1,134	953/1,017	0.054
Delay[♯] (IQR), day	1 (1–3)	1 (0–2)	<0.0001	2 (1–3)	1 (1–3)	<0.0001	1 (0–2)	1 (0–1)	0.0037
Clinical Symptom and Syndromes[§]									
Frequency of diarrhea (IQR), times	5 (4–6)	5 (4–7)	0.60	4 (3–6)	5 (4–6)	0.0091	5 (4–8)	5 (4–7)	0.016
Stool character									
Watery	4653 (80.00)	2070 (70.36)	<0.0001	2820 (80.90)	604 (62.14)	<0.0001	1833 (78.67)	1466 (74.42)	0.00046
Mushy	658 (11.31)	417 (14.17)	0.0011	336 (9.64)	150 (15.43)	<0.0001	322 (13.82)	267 (13.55)	0.87
Mucous	484 (8.32)	410 (13.94)	<0.0001	318 (9.12)	216 (22.22)	<0.0001	166 (7.12)	194 (9.85)	0.00070
Bloody stool	89 (1.53)	105 (3.57)	<0.0001	61 (1.75)	65 (6.69)	<0.0001	28 (1.20)	40 (2.03)	0.026
Others	78 (1.34)	67 (2.28)	0.00094	56 (1.61)	13 (1.34)	0.66	22 (0.94)	54 (2.74)	<0.0001
Vomiting	1,846 (31.74)	492 (16.72)	<0.0001	1,213 (34.80)	144 (14.81)	<0.0001	633 (27.17)	348 (17.66)	<0.0001
Fever	1,137 (19.55)	593 (20.16)	<0.0001	941 (26.99)	335 (34.47)	<0.0001	196 (8.41)	258 (13.10)	<0.0001
Respiratory symptoms	465 (7.80)	46 (1.56)	<0.0001	460 (13.20)	44 (4.53)	<0.0001	5 (0.21)	2 (0.10)	0.43
Dehydration	238 (4.09)	83 (2.82)	0.12	196 (5.62)	24 (2.47)	0.00059	42 (1.80)	59 (2.99)	0.0093
Neurologic symptoms	51 (0.88)	5 (0.17)	0.0015	36 (1.03)	3 (0.31)	0.073	15 (0.64)	2 (0.10)	0.017

Data are n (%) unless otherwise indicated. All viruses and bacteria tested cases were used to compare virus positive and bacterium positive cases. Virus positive cases were defined as any virus positive & all bacteria negative cases. Bacterium positive cases were defined as all viruses negative & any bacterium positive cases. [*]Chi square test for comparisons when virus positive *vs* bacterium positive cases. [♯]Wilcoxon test for comparisons when virus positive *vs* bacterium positive. [§]The logistic regressions were applied to adjust the age, sex, delay when tested for comparisons between virus positive and bacterium positive cases. All statistical tests were two-sided and $p<0.05$ was statistically significant. Respiratory symptoms: cough, expectoration, sore throat, pectoralgia, et al. Neurologic symptoms: convulsion, listlessness, lethargy, neck stiffness, et al. IQR=interquartile range.

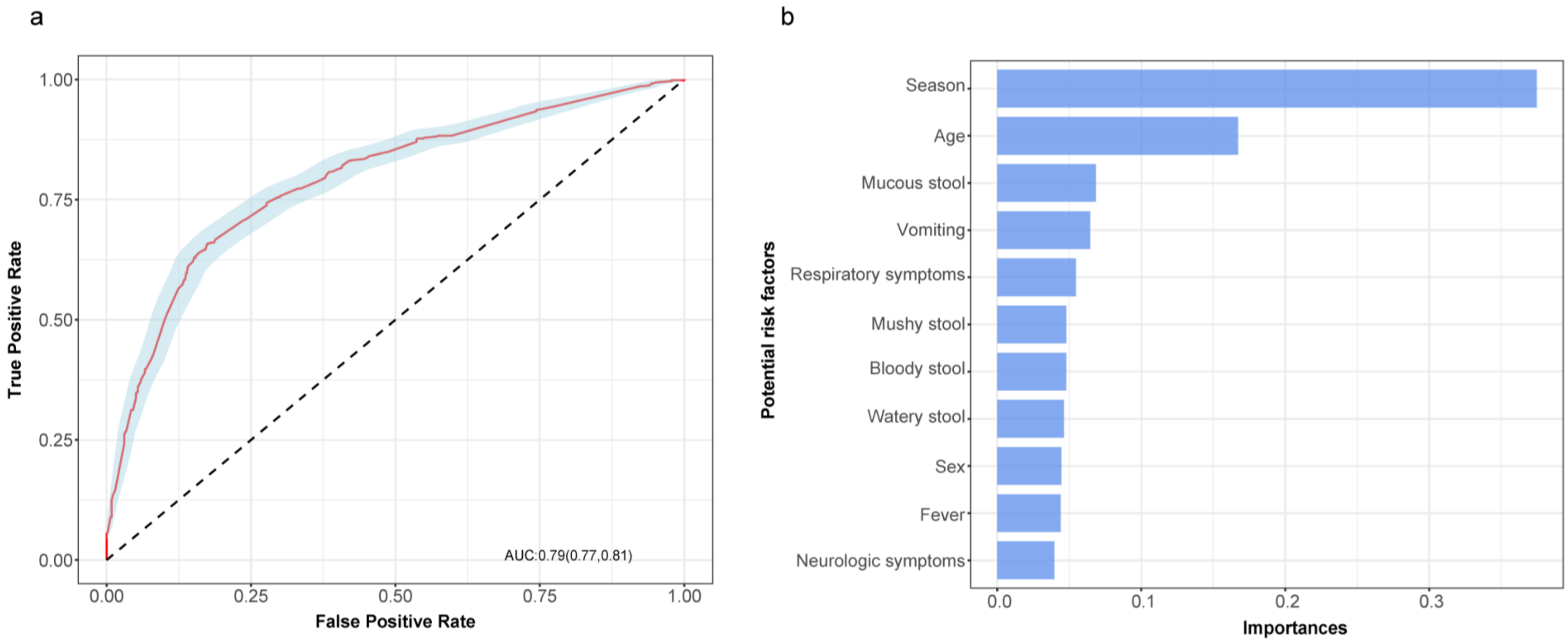

Supplementary Fig. 4: The predictive model for differentiation between viral infections and bacterial infections using demographic and clinical information by machine learning algorithms. (a) The red line is the ROC curve and the blue shading is the 95% confidence interval of the ROC. (b) The importance ranking of potential risk factors for binary XGBoost model to differentiate viral infections and bacterial infections. ROC=receiver operating characteristic. Source data are provided as a Source Data file.

Supplementary Table 10: Logistic regression analysis to distinguish the viral and bacterial infections (viral infections *vs* bacterial infections).

Variable	Univariate		Multivariate	
	Crude OR (95% CI)	*p*	Adjusted OR (95% CI)	*p*
Season				
Cold	1	-	1	-
Warm	0.12 (0.11, 0.14)	<0.001	0.13 (0.12, 0.15)	<0.001
Age group				
Children (<18 years old)	1	-	1	-
Adults (≥18 years old)	0.33 (0.30, 0.36)	<0.001	0.37 (0.33, 0.41)	<0.001
Sex				
Male	1	-	No significant and excluded	
Female	1.24 (1.14, 1.36)	<0.001		
Vomiting				
No	1	-	1	-
Yes	2.32 (2.07, 2.59)	<0.001	2.07 (1.83, 2.35)	<0.001
Watery stool				
No	1	-	No significant and excluded	
Yes	1.69 (1.52, 1.87)	<0.001		
Mucous stool				
No	1	-	1	-
Yes	0.56 (0.49, 0.64)	<0.001	0.50 (0.43, 0.59)	<0.001
Bloody stool				
No	1	-	1	-
Yes	0.42 (0.31, 0.56)	<0.001	0.38 (0.28, 0.53)	<0.001
Mushy stool				
No	1		No significant and excluded	
Yes	0.77 (0.68, 0.88)	<0.001		
Fever				
No	1	-		
Yes	0.96 (0.86, 1.08)	0.50		
Respiratory symptoms				
No	1	-	1	-
Yes	5.47 (4.07, 7.53)	<0.001	1.93 (1.40, 2.73)	<0.001
Neurologic symptoms				
No	1	-	No significant and excluded	
Yes	5.20 (2.28, 14.95)	<0.001		

Z test for comparisons among patients for different groups. All statistical tests were two-sided and $p<0.05$ was statistically significant. OR=odds ratio; 95% CI=95% confidence interval.

Supplementary Table 11: Factors associated with daily positive rate of enteropathogens evaluated by a multilevel negative binomial regression model at the sentinel city level in patients with acute diarrhea, China, 2009–2018.

	Rotavirus A		Norovirus		Adenovirus		Astrovirus		Sapovirus		Rotavirus B		Rotavirus C	
	IRR (95%CI)	*p*	IRR (95%CI)	*p*	IRR (95%CI)	*p*	IRR (95%CI)	*p*	IRR (95%CI)	*p*	IRR (95%CI)	*p*	IRR (95%CI)	*p*
Temperature (5 °C)	L0=0.83	<0.0001	L0=0.96	<0.0001	L9=1.04	0.00058	L10=0.87	<0.0001	L0=0.88	<0.0001	NS	-	#	-
	(0.82, 0.84)		(0.94, 0.97)		(1.02, 1.07)		(0.85, 0.89)		(0.85, 0.90)					
Sunshine duration (10 hour)	L0=0.76	<0.0001	NS	-	#	-	NS	-	NS	-	L2=0.64	0.02	L2=0.50	0.0012
	(0.72, 0.81)										(0.44, 0.94)		(0.33, 0.76)	
Precipitation (10 mm)	L3=0.91	<0.0001	L0=0.94	0.0024	NS	-	NS	-	NS	-	L8=0.78	0.046	NS	-
	(0.88, 0.94)		(0.91, 0.98)								(0.60, 0.98)			
Relative humidity (10 %)	NS	-	L10=1.05	<0.0001	#	-	L10=0.90	<0.0001	L0=0.96	0.039	NS	-	L9=0.84	<0.0001
			(1.03, 1.07)				(0.87, 0.93)		(0.92, 1.00)				(0.76, 0.93)	
Wind speed (m/s)	L4=0.83	<0.0001	NS	-	L9=0.91	0.0074	L2=0.90	0.0039	#	-	L7=0.74	0.0047	L6=0.73	0.011
	(0.81, 0.86)				(0.85, 0.98)		(0.83, 0.97)				(0.59, 0.92)		(0.57, 0.93)	
Air pressure (10 kPa)	#	-	L0=1.15	<0.0001	NS	-	L10=1.12	0.011	#	-	#	-	#	-
			(1.09, 1.21)				(1.02, 1.24)							
GDP *per capita* (10 thousand Yuan/person)	L0=1.04	<0.0001	#	-	NS	-	L0=0.90	<0.0001	NS	-	NS	-	NS	-
	(1.03, 1.06)						(0.88, 0.92)							
Population density (thousand people/km^2)	L0=0.83	<0.0001	L0=1.13	<0.0001	NS	-	NS	-	NS	-	NS	-	NS	-
	(0.80, 0.86)		(1.09, 1.17)											
Proportion of children (1 %)[§]	L0=1.03	<0.0001	L0=1.03	<0.0001	L0=1.05	<0.0001	NS	-	#	-	L0=1.10	<0.0001	L0=1.17	<0.0001
	(1.02, 1.04)		(1.02, 1.04)		(1.03, 1.06)						(1.06, 1.14)		(1.13, 1.21)	
Proportion of the older people (1 %)[‡]	L0=0.85	<0.0001	L0=1.10	<0.0001	L0=0.86	<0.0001	#	-	L0=1.17	<0.0001	L0=0.80	<0.0001	L0=0.91	0.04
	(0.84, 0.87)		(1.08, 1.12)		(0.84, 0.88)				(1.12, 1.23)		(0.74, 0.86)		(0.83, 0.99)	

Supplementary Table 11 continued

	DEC		NTS		*Shigella*		*V. parahaemolyticus*		*A. hydrophila*		*C. jejuni*		*V. cholerae*		*Y. enterocolitica*		*C. coli*	
	IRR (95%CI)	*p*	IRR (95%CI)	*p*	IRR (95%CI)	*p*	IRR (95%CI)	*p*	IRR (95%CI)	*p*	IRR (95%CI)	*p*	IRR (95%CI)	*p*	IRR (95%CI)	*p*	IRR (95%CI)	*p*
Temperature (5 ℃)	L9=1.26 (1.23, 1.28)	<0.0001	L6=1.26 (1.23, 1.29)	<0.0001	L9=1.31 (1.27, 1.36)	<0.0001	L5=1.70 (1.62, 1.78)	<0.0001	L6=1.30 (1.24, 1.37)	<0.0001	#	-	L4=1.28 (1.17, 1.42)	<0.0001	NS	-	#	-
Sunshine duration (10 hour)	L10=1.10 (1.01, 1.19)	0.031	NS	-	L8=0.77 (0.66, 0.91)	0.0018	L2=1.28 (1.09, 1.50)	0.0031	NS	-	L8=0.75 (0.59, 0.94)	0.011	NS	-	NS	-	#	-
Precipitation (10 mm)	NS	-	L6=1.04 (1.00, 1.08)	0.024	NS	-	NS	-	L3=1.09 (1.02, 1.17)	0.012	NS	-	NS	-	L3=0.74 (0.55, 0.95)	0.03	L7=0.66 (0.43, 0.93)	0.033
Relative humidity (10 %)	#	-	NS	-	L8=0.88 (0.85, 0.92)	<0.0001	L3=1.23 (1.17, 1.30)	<0.0001	NS	-	L4=1.17 (1.10, 1.25)	<0.0001	L1=1.40 (1.24, 1.58)	<0.0001	L6=0.86 (0.79, 0.94)	0.0015	NS	-
Wind speed (m/s)	NS	-	NS	-	L3=0.83 (0.77, 0.90)	<0.0001	L6=0.88 (0.81, 0.96)	0.0043	L1=0.84 (0.75, 0.94)	0.0022	NS	-	NS	-	L7=1.38 (1.16, 1.64)	<0.0001	#	-
Air pressure (10 kPa)	NS	-	L6=1.51 (1.40, 1.63)	<0.0001	NS	-	L0=3.54 (2.61, 4.93)	<0.0001	L0=1.65 (1.40, 1.96)	<0.0001	#	-	L0=0.62 (0.48, 0.80)	<0.0001	#	-	NS	-
GDP *per capita* (10 thousand Yuan/person)	NS	-	#	-	L0=0.82 (0.80, 0.83)	<0.0001	L0=1.35 (1.30, 1.39)	<0.0001	L0=0.93 (0.90, 0.96)	<0.0001	NS	-	#	-	#	-	NS	-
Population density (thousand people/km^2)	L0=1.14 (1.11, 1.18)	<0.0001	L0=1.11 (1.06, 1.16)	<0.0001	L0=0.82 (0.76, 0.88)	<0.0001	L0=0.73 (0.69, 0.77)	<0.0001	#	-	L0=2.44 (2.26, 2.65)	<0.0001	L0=0.49 (0.40, 0.61)	<0.0001	#	-	L0=2.19 (1.85, 2.62)	<0.0001
Proportion of children (1 %)[§]	NS	-	L0=1.04 (1.03, 1.05)	<0.0001	NS	-	NS	-	NS	-	NS	-	L0=0.86 (0.82, 0.90)	<0.0001	#	-	NS	-
Proportion of the older people (1 %)[‡]	NS	-	L0=0.89 (0.87, 0.91)	<0.0001	NS	-	L0=1.64 (1.54, 1.74)	<0.0001	NS	-	NS	-	#	-	L0=1.22 (1.09, 1.37)	0.00061	NS	-

Lx: the lagged days

[#]Variable had no significance and was excluded in univariate analysis; NS Variable had no significance and was excluded in multivariate analysis. NS=no significant.

[§]percentage of people younger than 14 years old

[‡]percentage of people older than 65 years old

Z test for comparisons among patients for different groups. All statistical tests were two-sided and $p<0.05$ was statistically significant. IRR=incidence rate ratio; 95% CI=95% confidence interval; DEC=diarrheagenic *Escherichia coli*; NTS=nontyphoidal *Salmonella*.

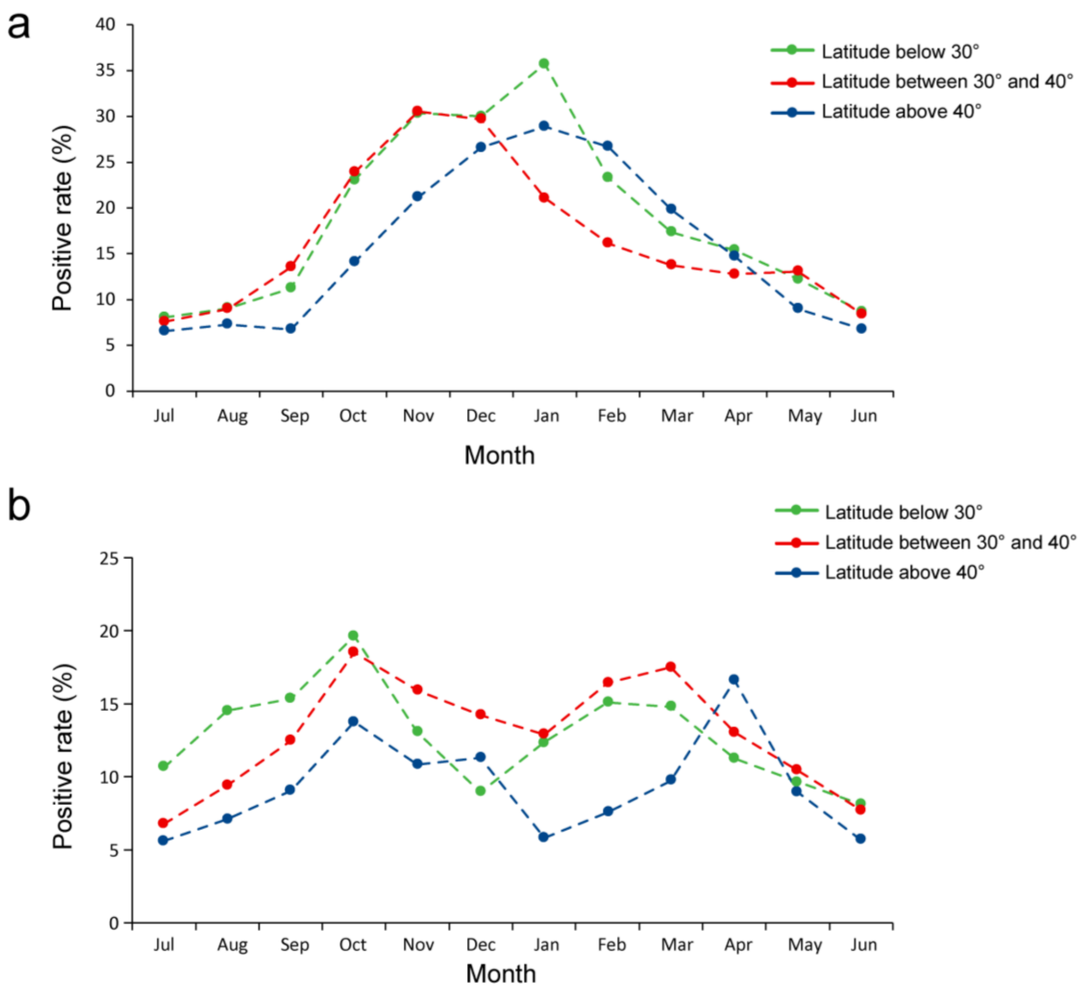

Supplementary Fig. 5: The seasonal pattern of rotavirus and norovirus at different latitudes. (a) The seasonal pattern of rotavirus. (b) The seasonal pattern of norovirus. Source data are provided as a Source Data file.

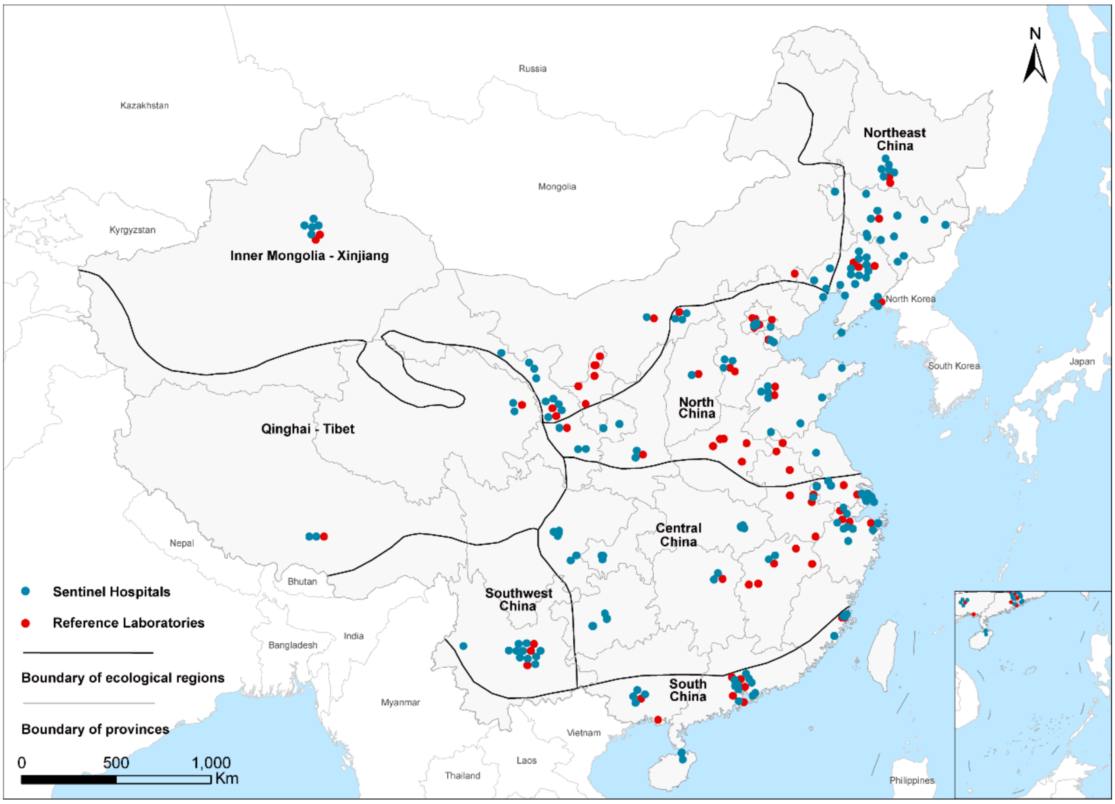

Supplementary Fig. 6: Location of 217 sentinel hospitals and 93 reference laboratories participating in diarrheal surveillance in the mainland of China, 2009–2018.

Each point indicates the position of a sentinel hospital (blue) or laboratory (red). The black lines present the delineation of seven ecological regions and the grey lines present the province boundaries. Northeast China has 36 sentinel hospitals and seven reference laboratories; North China has 33 sentinel hospitals and 26 reference laboratories; Inner Mongolia-Xinjiang has 19 sentinel hospitals and 12 reference laboratories; Qinghai-Tibet has four sentinel hospitals and two reference laboratories; Southwest China has 11 sentinel hospitals and three reference laboratories; Central China has 83 sentinel hospitals and 30 reference laboratories; South China has 31 sentinel hospitals and 13 reference laboratories. Source data are provided as a Source Data file.

Supplementary Table 12. Primers and sequence information for PCR used in diarrheal illnesses surveillance of China, 2009-2018.

Supplementary Table 12-1. Primers and sequence information used for characterizing G/P genotypes of Group A Rotavirus by RT-PCR.

	Genotype	Primer	Sequence (5'-3')*	Amplicon size (bp)	Position	Reference
G-typing	G	VP7F	ATGTATGGTATTGAATATACCAC	881	51-71	[9]
		VP7R	AACTTGCCACCATTTTTTCC		914-932	
	G1	aBT1	CAAGTACTCAAATCAATGATGG	618	314-335	[9, 10]
	G2	aCT2	CAATGATATTAACACATTTTCTGTG	521	411-435	[9, 10]
	G3	G3	ACGAACTCAACACGAGAGG	682	250-269	[9, 10]
	G4	aDT4	CGTTTCTGGTGAGGAGTTG	452	480-499	[9, 10]
	G8	aAT8	GTCACACCATTTGTAAATTCG	754	178-198	[9, 10]
	G9	G9	CTTGATGTGACTAYAAATAC	179	757-776	[9, 10]
P-typing	P	VP4F	TATGCTCCAGTNAATTGG	663	132-149	[11]
		VP4R	ATTGCATTTCTTTCCATAATG		775-795	
	P[4]	2T-1	CTATTGTTAGAGGTTAGAGTC	362	474-492	[11]
	P[6]	3T-1	TGTTGATTAGTTGGATTCAA	146	259-278	[11]
	P[8]	1T-1D	TCTACTGGRTTRACNTGC	224	339-356	[11]
	P[9]	4T-1	TGAGACATGCAATTGGAC	270	385-402	[11]
	P[10]	5T-1	ATCATAGTTAGTAGTCGG	462	575-594	[11]
	P[11]	P[11]	GTAAACATCCAGAATGTG	191	305-323	[11]

*N = any base; R = A or G; Y = C or T.

Supplementary Table 12-2. Primers and sequence information used in PCR for viral agents.

Pathogen	Primers and sequence information				
	Primer	Polarity*	Sequence (5'-3')#	Amplicon size (bp)	Reference
Rotavirus B	B5-2	+	GGCAATAAAATGGCTTCATTGC	814	[12]
	B3-3	-	GGGTTTTTACAGCTTCGGCT		
Rotavirus C	NG8S1	+	ATTATGCTCAGACTATCGCCAC	352	[13]
	NG8A2	-	GTTTCTGTACTAGCTGGTGAAC		
Adenovirus	Ad1	+	TTCCCCATGGCICAYAACAC	482	[13]
	Ad2	-	CCCTGGTAKCCRATRTTGTA		
Astrovirus	Mon269	+	CAACTCAGGAAACAGGGTGT	449	[14]
	Mon270	-	TCAGATGCATTGTCATTGGT		
Norovirus (genogroups I)	G1-SKF	+	CTGCCCGAATTYGTAAATGA	330	[15]
	GI-SKR	-	CCAACCCARCCATTRTACA		
Norovirus (genogroups II)	CoG2F	+	CARGARBCNATGTTYAGRTGGATGAG	387	[15]
	G2-SKR	-	CCRCCNGCATRHCCRTTRTACAT		
Sapovirus	SLV-5317	+	CTCGCCACCTACRAWGCBTGGTT	434	[15]
	SLV-5749	-	CGGRCYTCAAAVSTACCBCCCCA		

*"+" = forward primer; "-" = reverse primer;

#B = C, G, or T; H = A, C, or T; K = G or T; N = any base; R = A or G; S = G or C; V = A, C or G; W = A or T; Y = C or T.

Supplementary Table 12-3. Primers and sequence information used in amplification of diarrheagenic *Escherichia coli* genes.

Pathogen	Primer	Sequence (5'-3')	Amplicon size (bp)	Reference
EPEC	*eae*-F	TCAATGCAGTTCCGTTATCAGTT	482	[16]
	eae-R	GTAAAGTCCGTTACCCCAACCTG		
	bfp-F	GGAAGTCAAATTCATGGGGGTAT	300	[16]
	bfp-R	GGAATCAGACGCAGACTGGTAGT		
ETEC	*lt*-F	GCACACGGAGCTCCTCAGTC	218	[16]
	lt-R	TCCTTCATCCTTTCAATGGCTTT		
	*st*II-F	AAAGGAGAGCTTCGTCACATTTT	129	[16]
	*st*II-R	AATGTCCGTCTTGCGTTAGGAC		
EHEC	*eae*-F	TCAATGCAGTTCCGTTATCAGTT	482	[16]
	eae-R	GTAAAGTCCGTTACCCCAACCTG		
	*stx*1-F	CAGTTAATGTGGTGGCGAAGG	348	[16]
	*stx*1-R	CACCAGACAATGTAACCGCTG		
	*stx*2-F	ATCCTATTCCCGGGAGTTTACG	584	[16]
	*stx*2-R	GCGTCATCGTATACACAGGAGC		
EIEC	*virF*-F	AGCTCAGGCAATGAAACTTTGAC	618	[16]
	virF-R	TGGGCTTGATATTCCGATAAGTC		
	ipaH-F	CTCGGCACGTTTTAATAGTCTGG	933	[16]
	ipaH-R	GTGGAGAGCTGAAGTTTCTCTGC		
EAEC	*aaf*II-F	CACAGGCAACTGAAATAAGTCTGG	378	[16]
	*aaf*II-R	ATTCCCATGATGTCAAGCACTTC		

Supplementary Table 12-4. Primers and sequence information used in PCR for *C. jejuni* and *C. coli*.

Pathogen	Primer	Sequence (5'-3')	Amplicon size (bp)	Reference
C. jejuni	MapA-F	CTATTTTATTTTTGAGTGGTTGTG	589	[17]
	MapA -R	GCTTTATTTGCCATTTGTTTTATTA		
C. coli	CeuE-F	ATTTGAAAATTGCTCCAACTATG	462	[17]
	CeuE-R	TGATTTTATTATTTGTAGCAGCG		

Supplementary Table 12-5. Primers and sequence information used in amplification of *Y. enterocolitica* and *Y. pseudotuberculosis* genes.

Pathogen	Primer	Sequence (5'-3')	Amplicon size (bp)	Reference
Y. enterocolitica	*ail*-F	TAATGTGTACGCTGCGAG	351	[18]
	ail -R	GACGTCTTACTTGCACTG		
	ystA-F	ATCGACACCAATAACCGCTGAG	79	[18]
	ystA- R	CCAATCACTACTGACTTCGGCT		
	ystB-F	GTACATTAGGCCAAGAGACG	146	[18]
	ystB-R	GCAACATACCTCACAACACC		
	yadA-F	CTTCAGATACTGGTGTCGCTGT	849	[18]
	yadA-R	ATGCCTGACTAGAGCGATATCC	759*	
	virF-F	GGCAGAACAGCAGTCAGACATA	561	[18]
	virF- R	GGTGAGCATAGAGAATACGTCG		
Y. pseudotuberculosis	*inv*-F	CGGTACGGCTCAAGTTAATCTG	183	[18, 19]
	inv-R	CCGTTCTCCAATGTACGTATCC		
	yadA-F	CTTCAGATACTGGTGTCGCTGT	849	[18, 19]
	yadA-R	ATGCCTGACTAGAGCGATATCC		
	virF-F	TCATGGCAGAACAGCAGTCAG	590	[18, 19]
	virF- R	ACTCATCTTACCATTAAGAAG		
	ypmA-F	CACTTTTCTCTGGAGTAGCG	350	[18, 19]
	ypmA-R	ACTCATCTTACCATTAAGAAG		
	ypmB-F	TTTCTGTCATTACTGACATTA	453	[18, 19]
	ypmB-R	CCTCTTTCCATCCATCTCTTA		
	ypmC-F	ACACTTTTCTCTGGAGTAGCG	418	[18, 19]
	ypmC-R	ACAGGACATTTCGTCA		

*The amplicon of serotypes O:8 *Y. enterocolitica*

Supplementary Table 12-6. Primers and sequence information used in PCR for parasitical agents.

Pathogen	Gene	Primers and sequence information		
		Sequence (5’-3’)	Amplicon size (bp)	Reference
E. histolytica	30-kDa protein	GGAGGAGTAGGAAAGTTGAC<br>TTCTTGCAATTCCTGCTTCGA	100	[20]
G. lamblia	TPI	TPIF1: AAATIATGCCTGCTCGTCG<br>TPIR1: CAAACCTTITCCGCAAACC	605	[21]
		TPIF2: CCCTTCATCGGIGGTAACTT<br>TPIR2: GTGGCCACCACICCCGTGCC	530	[21]
Cryptosporidium	SSUrRNA	CryF1: TTCTAGAGCTAATACATGCG<br>CryR1: CCATTTCCTTCGAAACAGGA	1325	[22]
		CryF2: GGAAGGGTTGTATTTATTAGATAAAG<br>CryR2: CTCATAAGGTGCTGAAGGAGTA	826-864	[22]

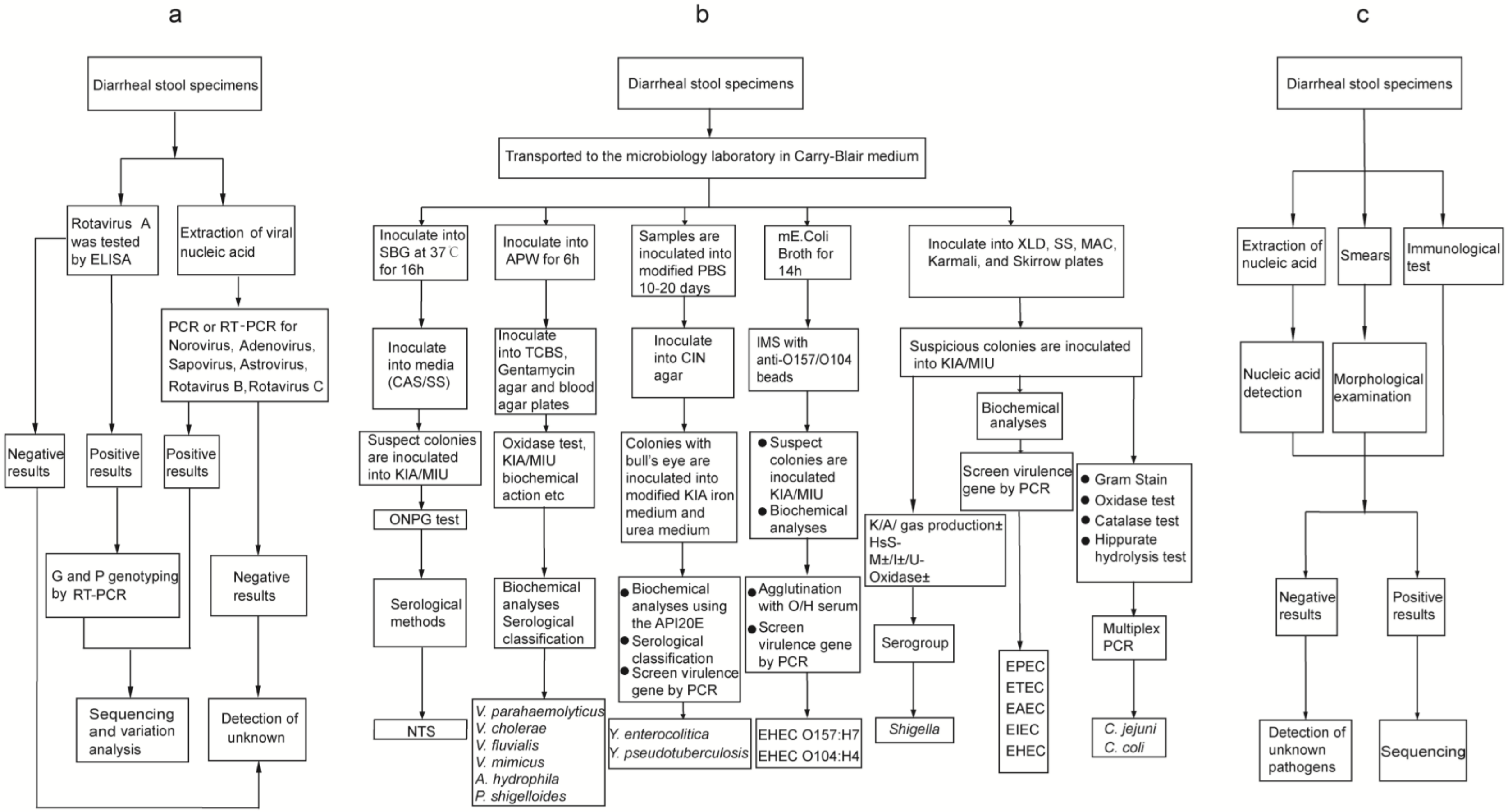

Supplementary Fig. 7: The flowchart of laboratory tests.

(a) Flow chart for viral pathogens test. (b) Flow chart for bacterial pathogens test. (c) Flow chart for parasitical pathogens test.

SBG=selenite brilliant green; APW=alkaline peptone water; PBS=phosphate buffered saline; XLD=xylose lysine desoxycholate; SS=*Salmonella-Shigella*; MAC=MacConkey; CAS=Chrom agar Salmonella; TCBS=thiosulfate-citrate-bile salts-sucrose; CIN=Cefsulodin-irgasan-novobiocin; KIA=Kligler iron agar; MIU=motility indole urea; ONPG=the ortho-nitrophenol test for beta-galactosidase production; DEC=diarrheagenic *Escherichia coli*; NTS=nontyphoidal *Salmonella*; EAEC=enteroaggregative *E. coli*; EPEC=enteropathogenic *E. coli*; ETEC=enterotoxigenic *E. coli*; EIEC=enteroinvasive *E. coli*; EHEC=enterohaemorrhagic *E. coli*.

Supplementary References

1. Yu, J. X. *et al.* Etiology of diarrhea among children under the age five in China: results from a five-year surveillance. *J. Infect.* **71**, 19-27 (2015).
2. Jing, H. Q., Huang, L. Y. & Duan, Z. J. Pathogen surveillance and detection techniques: diarrhea syndrome. (Sun Yat-Sen University Press, Guangzhou, 2016). (in Chinese)
3. Editorial board of encyclopedia of China. Encyclopedia of China-Chinese geography. (Encyclopedia of China Press, Beijing, 2004). (in Chinese)
4. National Bureau of Statistics. Available at: http://www.stats.gov.cn/tjsj/tjbz/tjyqhdmhcxhfdm/ (2020).
5. Kim, H. J., Fay, M. P., Feuer, E. J. & Midthune, D. N. Permutation tests for joinpoint regression with applications to cancer rates. *Stat. Med.* **19**, 335-351 (2000).
6. Available at: https://surveillance.cancer.gov/help/joinpoint/setting-parameters/method-and-parameters-tab/apc-aapc-tau-confidence-intervals/average-annual-percent-change-aapc (2020).
7. Nickbakhsh, S. *et al.* Virus-virus interactions impact the population dynamics of influenza and the common cold. *Proc. Natl. Acad. Sci. U. S. A.* **116**, 27142-27150 (2019).
8. Yang, S. G. *et al.* Epidemiological features of and changes in incidence of infectious diseases in China in the first decade after the SARS outbreak: an observational trend study. *Lancet Infect. Dis.* **17**, 716-725 (2017).
9. Iturriza Gómara, M. *et al.* Characterization of G10P[11] rotaviruses causing acute gastroenteritis in neonates and infants in Vellore, India. *J. Clin. Microbiol.* **42**, 2541-2547 (2004).
10. Gouvea, V. *et al.* Polymerase chain reaction amplification and typing of rotavirus nucleic acid from stool specimens. *J. Clin. Microbiol.* **28**, 276-282 (1990).
11. Simmonds, M. K. *et al.* New oligonucleotide primers for P-typing of rotavirus strains: Strategies for typing previously untypeable strains. *J. Clin. Virol.* **42**, 368-373 (2008).
12. Phan, T. G. *et al.* Development of a novel protocol for RT-multiplex PCR to detect diarrheal viruses among infants and children with acute gastroenteritis in Eastern Russia. *Clin. Lab.* **51**, 429-435 (2005).
13. Yan, H. N. *et al.* Development of RT-multiplex PCR assay for detection of adenovirus and group A and C rotaviruses in diarrheal fecal specimens from children in China. *Kansenshogaku Zasshi* **78**, 699-709 (2004).
14. Noel, J. S., Lee, T. W., Kurtz, J. B., Glass, R. I. & Monroe, S. S. Typing of human astroviruses from clinical isolates by enzyme immunoassay and nucleotide sequencing. *J. Clin. Microbiol.* **33**, 797-801 (1995).
15. Yan, H. N., Yagyu, F., Okitsu, S., Nishio, O. & Ushijima, H. Detection of norovirus (GI, GII), Sapovirus and astrovirus in fecal samples using reverse transcription single-round multiplex PCR. *J. Virol. Methods* **114**, 37-44 (2003).
16. Vidal, M. *et al.* Single multiplex PCR assay to identify simultaneously the six categories of diarrheagenic *Escherichia coli* associated with enteric infections. *J. Clin. Microbiol.* **43**, 5362-5365 (2005).
17. Denis, M. *et al.* Development of a m-PCR assay for simultaneous identification of *Campylobacter jejuni* and *C. coli*. *Lett. Appl. Microbiol.* **29**, 406-410 (1999).
18. Thoerner, P. *et al.* PCR detection of virulence genes in *Yersinia enterocolitica* and *Yersinia pseudotuberculosis* and investigation of virulence gene distribution. *Appl. Environ. Microbiol.* **69**, 1810-1816 (2003).
19. Carnoy, C. *et al.* The superantigen gene *ypm* is located in an unstable chromosomal locus of *Yersinia pseudotuberculosis*. *J. Bacteriol.* **184**, 4489-4499 (2002).
20. Fotedar, R. *et al.* Laboratory diagnostic techniques for *Entamoeba* species. *Clin. Microbiol. Rev.* **20**, 511-532 (2007).
21. Sulaiman, I. M. *et al.* Triosephosphate isomerase gene characterization and potential zoonotic transmission of *Giardia duodenalis*. *Emerg. Infect. Dis.* **9**, 1444-1452 (2003).
22. Xiao, L. & Fayer, R. *Cryptosporidium* and cryptosporidiosis. (CRC Press, London, 2007).
