# Supplementary material for: Etiological, epidemiological, and clinical features of acute diarrhea in China
Source: Nat Commun. 2021 Apr 29;12:2464. doi: 10.1038/s41467-021-22551-z (PMC8085116; doi:10.1038/s41467-021-22551-z)
Supplement: Supplementary file 3 — Reporting Summary [file 41467_2021_22551_MOESM3_ESM.pdf]

nature research

Corresponding author(s): Li-Qun Fang

Last updated by author(s): Mar 11, 2021

Reporting Summary

Nature Research wishes to improve the reproducibility of the work that we publish. This form provides structure for consistency and transparency in reporting. For further information on Nature Research policies, see our Editorial Policies and the Editorial Policy Checklist.

Statistics

For all statistical analyses, confirm that the following items are present in the figure legend, table legend, main text, or Methods section.

n/a	Confirmed	
x		The exact sample size (n) for each experimental group/condition, given as a discrete number and unit of measurement
x		A statement on whether measurements were taken from distinct samples or whether the same sample was measured repeatedly
	x	The statistical test(s) used AND whether they are one- or two-sided<br>*Only common tests should be described solely by name; describe more complex techniques in the Methods section.*
	x	A description of all covariates tested
	x	A description of any assumptions or corrections, such as tests of normality and adjustment for multiple comparisons
	x	A full description of the statistical parameters including central tendency (e.g. means) or other basic estimates (e.g. regression coefficient) AND variation (e.g. standard deviation) or associated estimates of uncertainty (e.g. confidence intervals)
	x	For null hypothesis testing, the test statistic (e.g. F, t, r) with confidence intervals, effect sizes, degrees of freedom and P value noted<br>*Give P values as exact values whenever suitable.*
x		For Bayesian analysis, information on the choice of priors and Markov chain Monte Carlo settings
x		For hierarchical and complex designs, identification of the appropriate level for tests and full reporting of outcomes
x		Estimates of effect sizes (e.g. Cohen's d, Pearson's r), indicating how they were calculated

Our web collection on statistics for biologists contains articles on many of the points above.

Software and code

Policy information about availability of computer code

Data collection	No software was used in the data collection.
Data analysis	R statistical software (version 3.5.3), SAS software version 9.4, ArcGIS software version 10.5, and Joinpoint Trend Analysis Software (version 4.7.0.0) were used in the data analysis. No custom code was used in this analyses.

For manuscripts utilizing custom algorithms or software that are central to the research but not yet described in published literature, software must be made available to editors and reviewers. We strongly encourage code deposition in a community repository (e.g. GitHub). See the Nature Research guidelines for submitting code & software for further information.

Data

Policy information about availability of data

All manuscripts must include a data availability statement. This statement should provide the following information, where applicable:

- Accession codes, unique identifiers, or web links for publicly available datasets
- A list of figures that have associated raw data
- A description of any restrictions on data availability

Relevant data that support the findings of this study and model results generated as part of this study are publicly available within the paper and its supplementary information files. Raw data are not publicly available due to restrictions by the data provider, which were used under license for the current study, but may be available from the authors upon reasonable request and permission from the data provider. All maps in this study are generated by the authors; no permission required for publication. Source data are provided with the paper.

Field-specific reporting

Please select the one below that is the best fit for your research. If you are not sure, read the appropriate sections before making your selection.

☐ Life sciences ☐ Behavioural & social sciences ☒ Ecological, evolutionary & environmental sciences

For a reference copy of the document with all sections, see nature.com/documents/nr-reporting-summary-flat.pdf

Ecological, evolutionary & environmental sciences study design

All studies must disclose on these points even when the disclosure is negative.

Study description	Based on a national surveillance network for patients with acute diarrhea, we made the first systematic attempt to identify the etiological, epidemiological, and clinical features of acute diarrhea in all-age population for a decade in China. It is anticipated that long-term continuous collection of surveillance data will be reasonably representative of the patients to a wide range and would be valuable for planning and adopting targeted preventive measures and therapy.
Research sample	Samples were recruited from patients with acute diarrhea administered in 217 sentinel hospitals of all 31 provinces (autonomous regions or municipalities) on the Chinese mainland. In this study, a case of acute diarrhea was defined as the presence of ≥3 passages of watery, loose, mucus-, or bloody-stools within a 24-h period. The rationale for the sample choice was that patients visiting clinics of sentinel hospitals that conform to the case definition of acute diarrhea. Patients referred from other hospitals or patients not initially diagnosed in sentinel hospitals or patients with non-infectious disease were excluded from this study. A random sampling method was used in sentinels to recruit participants. Our samples represent patients of acute diarrhea administered in hospital in all-age population in Chinese mainland.
Sampling strategy	No estimation of sample size was performed, as this was designed as a surveillance study to collect as many as possible patients that met the inclusion criteria, following the guideline of project.
Data collection	All participating hospitals used a surveillance protocol that included guidelines for patient enrollment, specimen collection and other related standard operating procedures that were developed by China CDC. Individual data about demography, clinical manifestations, laboratory testing results, medication use, and outcomes were collected by reviewing medical records and the data were entered into a standardized database by trained clinicians. All the data were uploaded to the online management system structured by the China CDC, sorted to remove redundant data, and checked for incomplete records.
Timing and spatial scale	Between January 2009 and December 2018, based on the National Key Science and Technology Project on Infectious Disease Surveillance Technique Platform, an active surveillance on patients with acute diarrhea was administered in 217 sentinel hospitals and 93 reference laboratories in all 31 province (autonomous regions or municipalities) in the Mainland of China. All the current data were obtained under this project framework. Thus the timing scale spanned from 1 January 2009 to 31 December 2018, with patients recruited in each monthly. The spatial scale covered all 31 provinces (autonomous regions or municipalities) in the Chinese mainland.
Data exclusions	Patients referred from other hospitals or patients not initially diagnosed in sentinel hospitals or patients with non-infectious disease were excluded from this study. Because our project gives priority to collect samples from patients who have not taken antibiotics when they were recruited, patients referred from other hospitals are highly likely been treated with medications, thus were excluded from the current study. All these criteria were pre-designed as guided by the SOP of the project.
Reproducibility	The analyses were performed independently three times in the last year, and all attempts to repeat the analyses were successful.
Randomization	Our study was based on a national surveillance network for patients with acute diarrhea, no experimental group and control were used.
Blinding	Our study was based on a national surveillance network for patients with acute diarrhea, blinding was not needed in the data collection.

Did the study involve field work? ☐ Yes ☒ No

Reporting for specific materials, systems and methods

We require information from authors about some types of materials, experimental systems and methods used in many studies. Here, indicate whether each material, system or method listed is relevant to your study. If you are not sure if a list item applies to your research, read the appropriate section before selecting a response.

Materials & experimental systems

n/a	Involved in the study	
☒	☐	Antibodies
☒	☐	Eukaryotic cell lines
☒	☐	Palaeontology and archaeology
☒	☐	Animals and other organisms
☐	☒	Human research participants
☒	☐	Clinical data
☒	☐	Dual use research of concern

Methods

n/a	Involved in the study	
☒	☐	ChIP-seq
☒	☐	Flow cytometry
☒	☐	MRI-based neuroimaging

Human research participants

Policy information about studies involving human research participants

Population characteristics	Patients with acute diarrhea administered in 217 sentinel hospitals of all 31 provinces (autonomous regions or municipalities) on the Chinese mainland. 152,792 patients were enrolled in the final analysis. Of these, 58.96% (90,093/152,792) were male, 55.63% (85,001/152,792) were aged <18 years and 11.02% (16,847/152,792) were the elderly aged ≥60 years.
Recruitment	Samples were recruited from patients with acute diarrhea administered in 217 sentinel hospitals of all 31 provinces (autonomous regions or municipalities) on the Chinese mainland. In this study, a case of acute diarrhea was defined as the presence of ≥3 passages of watery, loose, mucus-, or bloody-stools within a 24-h period. The number of sentinel hospitals in Western China was less than those in the densely populated regions, as there was a lower than average population size in this region. This limitation may have hindered a full understanding of the dynamic pattern of the diarrhea pathogens in this region.
Ethics oversight	Chinese Center for Diseases Control and Prevention.

Note that full information on the approval of the study protocol must also be provided in the manuscript.
